# Peroxisome Proliferator–Activated Receptor δ Suppresses the Cytotoxicity of CD8^+^ T Cells by Inhibiting RelA DNA-Binding Activity

**DOI:** 10.1158/2767-9764.CRC-24-0264

**Published:** 2024-10-14

**Authors:** Bo Cen, Jie Wei, Dingzhi Wang, Raymond N. DuBois

**Affiliations:** 1 Department of Biochemistry and Molecular Biology, Medical University of South Carolina, Charleston, South Carolina.; 2 Hollings Cancer Center, Medical University of South Carolina, Charleston, South Carolina.

## Abstract

The molecular mechanisms regulating CD8^+^ cytotoxic T lymphocytes (CTL) are not fully understood. Here, we show that the peroxisome proliferator–activated receptor δ (PPARδ) suppresses CTL cytotoxicity by inhibiting RelA DNA binding. Treatment of *Apc*^*Min/+*^ mice with the PPARδ agonist GW501516 reduced the activation of normal and tumor-associated intestinal CD8^+^ T cells and increased intestinal adenoma burden. PPARδ knockout or knockdown in CTLs increased their cytotoxicity against colorectal cancer cells, whereas overexpression of PPARδ or agonist treatment decreased it. Correspondingly, perforin, granzyme B, and IFNγ protein and mRNA levels were higher in PPARδ knockout or knockdown CTLs and lower in PPARδ overexpressing or agonist-treated CTLs. Mechanistically, we found that PPARδ binds to RelA, interfering with RelA–p50 heterodimer formation in the nucleus, thereby inhibiting its DNA binding in CTLs. Thus, PPARδ is a critical regulator of CTL effector function.

**Significance:**

Here, we provide the first direct evidence that PPARδ plays a critical role in suppressing the immune response against tumors by downregulating RelA DNA-binding activity. This results in decreased expression of perforin, granzyme B, and IFNγ. Thus, PPARδ may serve as a valuable target for developing future cancer immunotherapies.

## Introduction

Naïve CD8^+^ T cells can be activated via their T-cell receptor (TCR) by dendritic cells presenting cognate antigens. The TCR, consisting of variable αβ chains noncovalently associated with nonpolymorphic CD3 proteins, is crucial for signaling downstream pathways (1). TCR signaling alone results in a nonresponsive state (anergy) in which T cells are refractory to restimulation. Co-ligation of other cell surface receptors, such as CD28, provides additional signals required to avoid anergy and result in a productive T-cell activation (1). Following antigen–receptor–mediated activation, CD8^+^ T cells proliferate and differentiate into effector cells, such as cytotoxic T lymphocytes (CTL), which are key players in cancer immunotherapy (2, 3). CTLs defend against virally infected and malignant cells (2) by secreting death-inducing effector molecules like perforin, granzymes, and Fas-ligand, as well as chemokines and effector cytokines like IFNγ and TNFα (2).

Peroxisome proliferator–activated receptors (PPAR) are nuclear hormone receptors that regulate the expression of multiple genes. PPARs influence T-cell survival, activation, and CD4^+^ T helper cell differentiation (4). PPARα and PPARγ agonists have shown promise in enhancing T-cell therapies by modulating metabolic pathways. Adaptive immune responses of T and B cells have been studied in animal models using genetic manipulation and by activating the receptors with synthetic ligands. Treatment with the PPARα agonist, fenofibrate, improved the efficacy of CD8^+^ T-cell therapy for melanoma in a patient-derived xenograft mouse model, likely through switching from glycolysis to fatty acid oxidation (5). Bezafibrate, a PPARγ agonist, improved the efficacy of PD-1 blockade by promoting differentiation of naïve to effector T cells, upregulating fatty acid oxidation, and inhibiting apoptosis of effector T cells (6). PPARδ (also known as PPARβ) plays a multifaceted role in cancer (7–9). As a transcription factor, PPARδ directly binds to peroxisome proliferator responsive elements (PPRE) within the promoters of target genes as a heterodimer with retinoid X receptor (7, 8). PPARδ represses some genes indirectly through interactions with other transcription factors (e.g., NF-κB) or transcriptional repressors such as B-cell lymphoma 6 that do not require DNA binding (7, 10). PPARδ can impact T-cell development and function (4) and protect activated human CD3^+^ T cells from undergoing apoptosis (11). A recent study implicated PPARδ as an additional regulator of a metabolic program that supports the growth of thymocytes and mature CD4^+^ T cells (12). The GOT2–PPARδ axis promotes spatial restriction of CD4^+^ and CD8^+^ T cells from the tumor microenvironment in a pancreatic cancer mouse model (13). The underlying mechanisms by which PPARδ exerts its actions in T cells are poorly elucidated, and it is not clear whether PPARδ directly regulates the cytotoxicity of CD8^+^ T cells.

The NF-κB family, including RelA (p65), RelB, c-Rel, NF-κB1 (p50), and NF-κB2 (p52), is crucial for innate and adaptive immune responses and TCR signaling (14). NF-κB is essential for T-cell development, survival, and effector differentiation (15). RelA plays a vital role for regulatory T-cell activation and stability (16). Mice lacking IKKβ were unable to reject subcutaneously injected tumors that wild-type mice otherwise eliminated (17). Anergic CD8^+^ T cells have impaired NF-κB activation, with defects in RelA phosphorylation and acetylation (18). Effector molecules like perforin, granzyme B, Fas-ligand, IFNγ, and TNFα, needed for cell killing, are targets of RelA (19–25). CTLs are potent effectors in many anticancer immune responses and are critical for the currently successful immunotherapies. This study investigates how PPARδ modulates CTL cytolytic activity.

## Materials and Methods

### Animals

All animal experiments were conducted in accordance with our animal protocols, approved by the Institutional Animal Care and Use Committee at MUSC. *Apc*^*Min/+*^ mice were obtained from The Jackson Laboratory. PPARδ null *Apc*^*Min/+*^ mice (*Ppard*^*−/−*^/*Apc*^*Min/+*^) and their control mice (*Ppard*^*+/+*^/*Apc*^*Min/+*^) were generated from the same litter mates by breeding *Ppard*^*−/−*^/*Apc*^*+/+*^ on a mixed genetic background (C57BL/6 × 129/SV) with *Ppard*^*+/+*^/*Apc*^*Min/+*^ on a C57BL/6 genetic background (The Jackson Laboratory) as described (26). PPARδ was deleted in the whole organism by deleting exons 4 to 5. Male mice were used for isolation of splenic CD8^+^ T cells.

### Reagents and antibodies

A PPARδ agonist GW501516 was obtained from Ramidus AB. A second PPARδ agonist GW0742 was obtained from Tocris. The following antibodies were purchased from Cell Signaling Technology: anti-cleaved PARP (#5625, RRID: AB_10699459), anti–cleaved caspase 7 (#9491, RRID: AB_2068144), anti–cleaved caspase 3 (#9664, RRID: AB_2070042), anti–granzyme B (#17215, RRID: AB_2798780), anti-GAPDH (#8884, RRID: AB_11129865), anti-perforin (#62550, RRID: AB_3095060), anti-perforin (mouse-specific; #44865), and anti–NF-κB1/p50 (#13586, RRID: AB_2665516). Anti–β-actin (#A3854, RRID: AB_262011) antibody was purchased from Sigma. Anti-RelA (#SC-372, RRID: AB_632037), anti-PPARδ (#SC-74517, RRID: AB_1128604), and anti–lamin A (#SC56137, RRID: AB_2136168) antibodies were obtained from Santa Cruz Biotechnology. Anti-IFNγ (#MM700B) antibody was purchased from Invitrogen. Anti-CPT1A (#A5307, RRID: AB_2766119) and anti-PGC1α (#A19674, RRID: AB_2862726) antibodies came from ABclonal Science. Horseradish peroxidase–linked enhanced chemiluminescence mouse (#NA934) and rabbit IgG (#NA931) were purchased from GE Healthcare Life Sciences. A GFP expression plasmid pmaxGFP was obtained from Lonza. The pcDNA3–human PPARδ plasmid was kindly provided by Dr. Imad Shureiqi (University of Michigan). Two siRNAs targeting human PPARδ (#4390826 and #4390825) were purchased from Ambion.

### Isolation of immunocytes from intestines

All fat and Peyer’s patches were removed from excised intestines under a dissecting microscope for intestinal immune cell preparation. Mouse normal intestinal tissues and adenomas were minced and digested with RPMI 1640 medium containing 5% FBS, 1 mmol/L MgCl_2_, 1 mmol/L CaCl_2_, 2.5 mmol/L HEPES, and 200 U/mL collagenase I (Gibco). The immune cells from intestinal tissues were enriched by using a discontinuous (44% and 67%) Percoll (GE) separation method. Isolated immune cells were subjected to flow cytometry.

### Flow cytometry analysis

For carboxyfluorescein diacetate succinimidyl ester (CFSE) cell proliferation, immune cells isolated from normal intestinal tissues or tumors were labeled with 0.5 μmol/L CFSE. CFSE-labeled immune cells were cultured in RPMI 1640 medium with 10% FBS for 24 hours. Then, these cells were incubated with the following antibodies in staining buffer at the following dilution for 30 minutes on ice: CD45-PE-Cy7 (1:250, BioLegend, Cat. #103114, RRID: AB_312979), CD8-PE (1:50, BioLegend, Cat. #100706, RRID: AB_312745), CD4-AF700 (1:100, BioLegend, Cat. #100536, RRID: AB_493701), CD3–PerCP 5.5 (1:100, Invitrogen, Cat. #45-0031-82), and V450 (1:1,500, Invitrogen, Cat. #65-0863-14). To analyze INFγ expression on CD8^+^ T cells, intestinal immune cells were stained with cell surface markers as described above. Then, the cells were fixed and permeabilized using a Cytofix/Cytoperm kit (BD Biosciences, Cat. #554714) followed by intracellular cellular staining with antimouse INFγ-FITC antibody (1:50, BD Biosciences, Cat. #554411, RRID: AB_395375) in permeabilization buffer for 30 minutes on ice. After incubation with antibodies, the cells were analyzed on a Fortessa X-20 cytometer (BD Biosciences). Dead cells were excluded using V450 staining. The flow cytometric profiles were analyzed by counting 30,000 events using FlowJo X software (Tree Star, RRID: SCR_008520).

### Cell culture and transfection

Colon cancer cell lines LS174T, HCA7, HT29 (RRID: CVCL_0320), and HCT116 (RRID: CVCL_0291) were secured from the ATCC. All cell lines were authenticated by providers utilizing short tandem repeat profiling. Cells were grown in McCoy’s 5A medium with L-glutamine (Corning Cellgro, #10-050-CV) and 10% FBS (GE Healthcare, #SH30071.3) at 37°C under 5% CO_2._ According to the manufacturer's instructions, human CD8^+^ T cells were isolated from frozen human peripheral blood mononuclear cells using a MojoSort Human CD8 T Cell Isolation Kit (BioLegend, #480012). Cells were activated and expanded by Dynabeads Human T-Activator CD3/CD28 (Thermo Fisher Scientific, #11161D) in ImmunoCult-XF medium (STEMCELL Technologies) in the presence of 50 IU/mL human recombinant IL2 (PeproTech, #200-02). Murine CD8^+^ T cells were isolated from spleens from wild-type (*Ppard*^*+/+*^) and PPARδ-null (*Ppard*^*−/−*^) mice using a mouse CD8a^+^ T Cell Isolation Kit, (Miltenyi Biotec, #130-104-075) according to the manufacturer’s instructions. Cells were activated and expanded by Dynabeads Mouse T-Activator CD3/CD28 (Thermo Fisher Scientific, #11452D) in ImmunoCult-XF medium (STEMCELL Technologies) in the presence of 50 U/mL human recombinant IL2 (PeproTech, #200-02). Plasmids or siRNAs were transfected into T cells with Amaxa Human or Mouse T Cell Nucleofector Kit using a Nucleofector II device (Lonza).

### Immunoblotting

Cells were harvested in lysis buffer consisting of 50 mmol/L Tris pH 7.4, 150 mmol/L NaCl, 1% NP-40, and 5 mmol/L EDTA. Following 30-minute incubation in lysis buffer at 4°C, lysates were cleared by centrifugation at 16,000 × *g* for 10 minutes at 4°C, and then protein concentrations were determined by DC Protein Assay (Bio-Rad). Peroxidase conjugated donkey antirabbit and sheep antimouse (1:10,000; GE Healthcare NA934 and NA931, respectively) antibodies were incubated for 1 hour at room temperature. ECL prime kit (GE Healthcare) was used to detect chemiluminescence on an Azure Imaging System C300 (Azure Biosystems). Densitometry analyses were performed using the ImageJ software (1.54 g, RRID: SCR_003070).

### *In vitro* cytotoxicity assay

For human or mouse colon tumor cells, human or mouse CTLs were cocultured, respectively, with 2 × 10^4^ indicated epithelial cells in the 96-well round-bottomed plates at ratios (E:T = 2:1) for 18 hours. The cytotoxicity of CTLs against tumor cells was measured using a CytoTox 96 Non-Radioactive Cytotoxicity Assay (Promega) to measure lactate dehydrogenase (LDH) release according to the manufacturer’s instructions. Percent cytotoxicity was calculated by the following formula: percent cytotoxicity = 100 × experimental LDH release (OD_490_)/maximum LDH release (OD_490_). The maximum LDH release was defined as the value of OD_490_ from cells incubated with lysis solution.

### Dual-Luciferase Reporter Assay

Plasmids or siRNAs were transfected into human or mouse CTLs with Amaxa Human or Mouse T cell Nucleofector Kit using a Nucleofector II device (Lonza). NF-κB firefly luciferase reporter was driven by a 5X NF-κB responsive element inserted into the Cis-reporter backbone (Stratagene). pRL-SV40 (Renilla luciferase, Promega) was used as a control. After transfection, cells were treated with 1 μmol/L GW501516 as indicated for 24 hours. Cells were lysed using cell lysis buffer provided in the kit (Promega, catalog no. E1960). Luciferase activity was measured using a Dual-Luciferase Reporter Assay kit (Promega) with a Monolight 3010 luminometer (BD Biosciences/Pharmingen). The relative luciferase activity was determined and normalized to Renilla luciferase activity.

### Measurement of RelA and p50 DNA-binding activity

The DNA-binding capacity of nuclear or purified RelA and p50 was quantitatively measured using Active Motif’s TransAM NF-κB p65 Kit (#40096), following the vendor’s instructions.

### Nuclear extraction

Nuclear extracts were obtained using Abcam’s Nuclear Extraction Kit (#ab113474) according to the vendor’s instructions.

### Coimmunoprecipitation and GST pulldown assay

All coimmunoprecipitation experiments were performed using nuclear extracts isolated from CTLs. The nuclear extracts were diluted in a coimmunoprecipitation buffer (20 mmol/L HEPES, pH 7.5, 150 mmol/L NaCl, 1% Triton X-100, 1 mmol/L EDTA, and 10% glycerol) and precleared with protein A/G agarose (Pierce, #20421) before incubation with anti-p65 (Cell Signaling Technology, #8242, RRID: AB_10859369) or anti-PPARδ (Santa Cruz, #SC-74517, RRID: AB_1128604 ) antibody and protein A/G agarose at 4°C overnight.

GST pulldown experiments were performed with purified proteins. A GST-tagged NF-κB1 p50 protein was incubated with p65 or PPARδ or both in coimmunoprecipitation buffer described above in the presence of Glutathione Sepharose 4B (Amersham, #17-0756-01) at 4°C for 3 hours. The beads from GST pulldown or coimmunoprecipitation experiments were centrifuged and washed three times with the coimmunoprecipitation buffer. After centrifugation, the pellet was resuspended in 1× SDS-PAGE sample buffer and boiled for 5 minutes. The samples were then subjected to Western blot analysis.

### Electrophoreticmobility shift assay

Electrophoreticmobility shift assay (EMSA) was performed using a LightShift EMSA Optimization and Control Kit (Thermo Fisher Scientific, #20148X) following the manufacturer’s protocol. Nuclear extracts (5 μg) from human CTLs were incubated with NF-κB oligonucleotide (5′-AGT​TGA​GGG​GAC​TTT​CCC​AGG​C-3′, Rockland, #K-025) and electrophoresed on a 4% nondenaturing polyacrylamide gel and stained with SYBR Green using EMSA kit (Thermo Fisher Scientific, #E33075). After staining, proteins were transferred to polyvinylidene difluoride membranes for Western blot analyses using antibodies against RelA, p50, or PPARδ.

### ELISA

The levels of IFNγ in CTL cell culture medium were determined using R&D Systems’ Human IFN-gamma DuoSet ELISA (#DY285B-05) or Mouse IFN-gamma DuoSet ELISA (#DY485-05) kits, following the vendor’s instructions.

### Chromatin immunoprecipitation–qPCR assay

Chromatin immunoprecipitation (ChIP) was performed using a ChIP assay kit (Upstate USA, Inc.). Briefly, the indicated cells were treated with 1% formaldehyde-containing medium for 10 minutes at 37°C to crosslink proteins to DNA. Crosslinked chromatins were sonicated to reduce the DNA length to 200 to 1,000 bp. At this point, samples of total chromatin were taken as a positive control (input chromatin). The cell lysates were precleared by incubation with Protein G-Sepharose beads and then incubated with an anti-p65 (Cell Signaling, #8242, RRID: AB_10859369) antibody or anti–acetyl-Histone H3 (Lys27) antibody (Cell Signaling, #8173, RRID: AB_10949503) overnight at 4°C. DNA–protein complexes were collected with Protein G-Sepharose beads followed by several rounds of washing, eluted, and reverse cross-linked. Following treatment with Protease K, the samples were extracted with phenol chloroform and precipitated with ethanol. The recovered DNA was resuspended in Tris-HCl-EDTA buffer and used for the qPCR amplification. The primer pairs amplify regions close to the transcription start sites. Acetyl-Histone H3 (Lys27) binding was used as a positive control. Samples were tested using the 3′-untranslated region (3′-UTR) primer pairs. The primer sequences are as follows: *Ifng*-forward, 5′-GCT GAG ATT ACA GGC ATA CAC C-3′, *Ifng*-reverse, 5′-AGC ACT TTG GGA GGT TGA G-3′; *Prf1*-forward, 5′-CAT AAG CCC CTG TTC CTG TAA G-3′, *Prf1*-reverse, 5′-TCT CAT GGG TCA CAC TTT GG-3′; *Gzmb*-forward, 5′-GTT GCC TCA CCC AGA AAG T-3′, *Gzmb*-reverse, 5′-TGG TGT CTG CCC AAA TAG C-3′. The primer sequences for the 3′-UTR regions are as follows: *Ifng*-forward, 5′-GCT TTA ATG GCA TGT CAG ACA G-3′, *Ifng*-reverse, 5′-TTG GGT ACA GTC ACA GTT GTC-3′; *Prf1*-forward, 5′-TGG TGA GAA CAG TGA GCT TG-3′, *Prf1*-reverse, 5′-AAT GGG AAT ACG AAG ACA GCC-3′; *Gzmb*-forward, 5′-ACA GGA AGC AAA CTA AGC CC-3′, *Gzmb*-reverse, 5′-CAC CTC TCC CAG TGT AAA TCT G-3′.

### RNA and qPCR

Total RNA was isolated from cultured cells using the RNeasy Mini Kit (Qiagen, #74106) and was reverse transcribed to cDNA using iScript cDNA Synthesis Kit (Bio-Rad, #1708891). qRT-PCR was performed with iQ SYBR Green Supermix (Bio-Rad, #1706682) using QuantStudio 7 Flex Real-time PCR System (Life Technologies). Primers were synthesized by Integrated DNA Technologies. The sequences of the specific PCR primers are as follows:

human *Prf1*-forward: 5′-GGA GTG CCG CTT CTA CAG-3′,

human *Prf1*-reverse: 5′-CGT AGT TGG AGA TAA GCC TGA G-3′;

mouse *Prf1*-forward: 5′-CAG TAG AGT GTC GCA TGT ACA G-3′,

mouse *Prf1*-reverse: 5′-GAG ATG AGC CTG TGG TAA GC-3′;

human *Gzmb*-forward: 5′-GTA CCA TTG AGT TGT GCG TG-3′,

human *Gzmb*-reverse: 5′-CAT GCC ATT GTT TCG TCC ATA G-3′;

mouse *Gzmb*-forward: 5′-CCT CCA GGA CAA AGG CAG-3′,

mouse *Gzmb*-reverse: 5′-CAG TCA GCA CAA AGT CCT CTC-3′;

human *Ifng*-forward: 5′-GCA TCG TTT TGG GTT CTC TTG-3′,

human *Ifng*-reverse: 5′-AGT TCC ATT ATC CGC TAC ATC TG-3′;

mouse *Ifng*-forward: 5′-CCT AGC TCT GAG ACA ATG AAC G-3′,

mouse *Ifng*-reverse: 5′-TTC CAC ATC TAT GCC ACT TGA G-3′;

human *Ppard*-forward: 5′-GCT TCC ACT ACG GTG TTC ATG-3′,

human *Ppard*-reverse: 5′-CTT CTC GTA CTC CAG CTT CAT G-3′;

mouse *Tnfa-*forward: 5′-CTT CTG TCT ACT GAA CTT CGG G-3′,

mouse *Tnfa*-reverse: 5′-CAG GCT TGT CAC TCG AAT TTT G-3′.

### Statistical analysis

Two independent *in vivo* experiments were conducted using three mice for each group in each experiment. ANOVA with two factors and two modalities were used to compare outcomes among multiple groups of mice. Factor 1 is an experiment with two modalities (experiment 1 and experiment 2), and factor 2 is treatment with two modalities (control and GW501516). Each *in vitro* experiment was done at least three times. Unpaired two-tailed Student *t* tests were used to assess the difference between the mean ± SD of the two groups. *P* values were considered significant at *, *P* < 0.05; **, *P* < 0.02.

### Data availability

Data were generated by the authors and available on request.

## Results

### PPARδ inhibits the proliferation and IFNγ expression of CD8^+^ T cells *in vivo* and the cytotoxicity of CD8^+^ T cells *in vitro*

**Figure 1:**
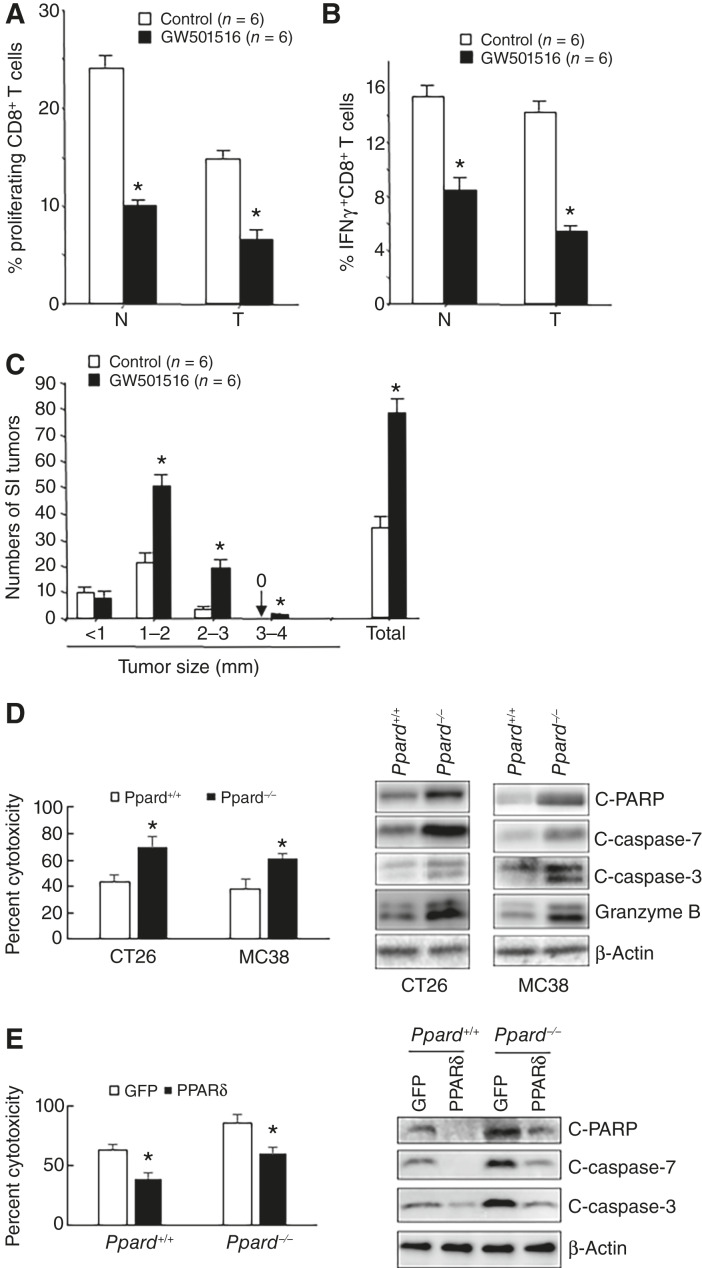
PPARδ inhibits CD8^+^ T-cell activation *in vivo* and cytolytic activity *in vitro*. **A,** Proliferation of CFSE-labeled CD8^+^ T cells from small intestine tumors and adjacent normal tissues in control and GW501516-treated mice. **B,** Percentage of IFNγ-expressing CD8^+^ T cells in small intestine tumor-specific and adjacent normal in control and GW501516-treated mice by flow cytometry. **C,** Number of small intestine tumors in different size groups in control and GW501516-treated mice. For **A–C**, the results were obtained from two independent experiments using three mice for each group in each experiment. **D,** The cytotoxicity of *Ppard*^*+/+*^ and *Ppard*^*−/−*^ murine CTLs (18-hour incubation, left) and Western blot expression of indicated proteins in mouse colorectal cancer cell lines CT26 and MC38 cells (2-hour incubation, right) after CTLs were incubated with colorectal cancer cells. Data (mean ± SD) represent three independent experiments with similar results. *, *P* < 0.05. **E,** The cytotoxicity of *Ppard*^*+/+*^ and *Ppard*^*−/−*^ murine CTLs (18-hour incubation, left) and Western blot expression of indicated proteins in CT26 cells (2-hour incubation, right) after CTLs transfected with plasmid expressing GFP or human PPARδ were incubated with CT26 cells. Data (mean ± SD) represent three independent experiments with similar results. *, *P* < 0.05. C-PARP, cleaved PARP; C-caspase 3, cleaved caspase 3; C-caspase 7, cleaved caspase 7.

We first examined the role of PPARδ in CD8^+^ T-cell regulation by treating male *Apc*^*Min/+*^ mice with a PPARδ agonist (GW501516). GW501516 treatment significantly decreased CD8^+^ T -cell proliferation (Fig. 1A) and IFNγ-expressing CD8^+^ T cells (Fig. 1B) in intestinal tumors and matched normal tissues compared with the control group, suggesting PPARδ regulates CD8^+^ T-cell activation. GW501516 treatment also significantly increased tumor burden (Fig. 1C).

To evaluate the role of PPARδ in CD8^+^ T-cell cytotoxicity, we isolated splenic CD8^+^ T cells from wild-type (*Ppard*^*+/+*^) and PPARδ-null (*Ppard*^*−/−*^) mice. CD8^+^ T cells were first stimulated with magnetic αCD3/CD28 beads and IL2 to become CTLs and then coincubated with murine colorectal cancer cell lines (CT26 or MC38). In this coculture system, activated CD8^+^ T cells recognize antigens presented in the context of cancer cell MHC class I molecules (27), allowing us to measure their cytotoxicity. *Ppard*^*−/−*^-CD8^+^ T cells showed increased cytotoxicity compared with wild-type *Ppard*^+/+^ CD8^+^ T cells [Fig. 1D (left)]. Cancer cells cocultured with *Ppard*^*−/−*^ CTLs had higher expression of apoptotic markers (cleaved PARP, cleaved caspase 7, and cleaved caspase 3) than those cocultured with wild-type cell CTLs [Fig. 1D (right); Supplementary Fig. S1A]. Overexpression of PPARδ in wild-type and *Ppard*^*−/−*^ CTLs inhibited their cytotoxicity and the expression of apoptotic markers (Fig. 1E; Supplementary Fig. S1B). These results demonstrate that PPARδ is capable of regulating CD8^+^ T-cell cytotoxicity. In human CTLs, PPARδ knockdown with two different siRNAs increased the killing of HCT116 cells and the expression of apoptotic markers (Supplementary Fig. S1C). In contrast, overexpression of PPARδ decreased the killing of HCT116 cells and apoptotic marker expression (Supplementary Fig. S1D). Additionally, GW501516 and GW0742, two unique PPARδ agonists suppressed the cytotoxicity of CTLs and the expression of apoptotic markers (Supplementary Fig. S1E). Thus, PPARδ negatively regulates CD8^+^ T-cell killing of tumor cells.

### PPARδ downregulates the expression of perforin, granzyme B, and IFNγ in CTLs

**Figure 2:**
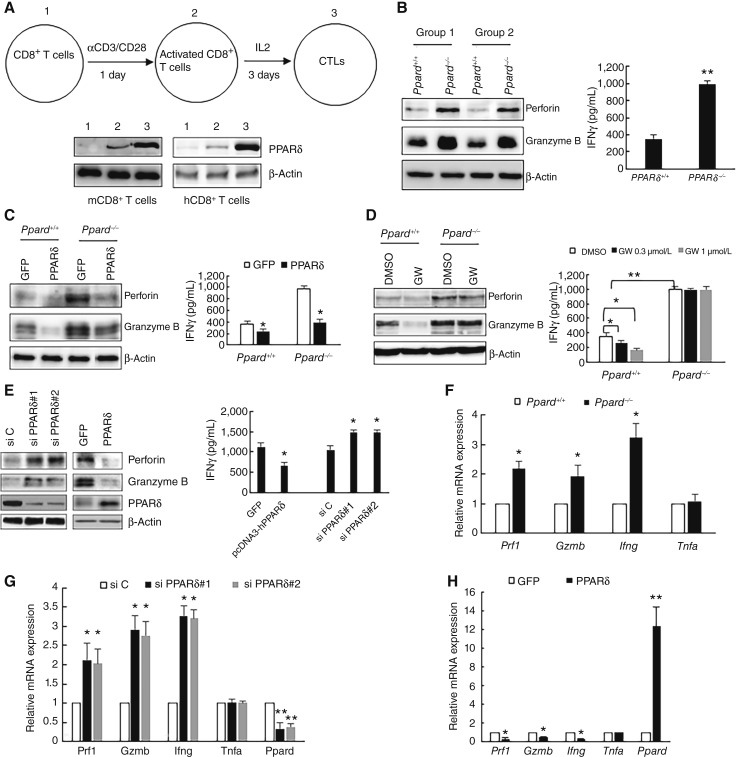
PPARδ negatively regulates the expression of perforin, granzyme B, and IFNγ. **A,** Western blot expression of PPARδ in naive, activated, and cytotoxic murine or human CD8^+^ T cells. **B,** Western blot expression of indicated proteins in *Ppard*^*+/+*^ and *Ppard*^*−/−*^ murine CTLs from two groups (two mice for each phenotype in a group) of mice and ELISA expression of IFNγ in the culture media of the CTLs. Data (mean ± SD) represent three independent experiments with similar results. **, *P* < 0.02. **C,** Western blot expression of indicated proteins in *Ppard*^*+/+*^ and *Ppard*^*−/−*^ murine CTLs transfected with a plasmid expressing GFP or human PPARδ and ELISA expression of IFNγ in the culture media of the CTLs. Data (mean ± SD) represent three independent experiments with similar results. *, *P* < 0.05. **D,** Western blot expression of indicated proteins in *Ppard*^*+/+*^ and *Ppard*^*−/−*^ murine CTLs treated with 1 μmol/L GW501516 (GW) and ELISA expression of IFNγ in the culture media of GW501516-treated CTLs. Data (mean ± SD) represent three independent experiments with similar results. *, *P* < 0.05; **, *P* < 0.02. **E,** Western blot expression of indicated proteins in human CTLs transfected with either two different PPARδ siRNAs or a plasmid expressing human PPARδ and ELISA expression of IFNγ in the culture media of the CTLs. Data (mean ± SD) represent three independent experiments with similar results. *, *P* < 0.05. **F,** The mRNA levels of indicated genes in the *Ppard*^*+/+*^ and *Ppard*^*−/−*^ murine CTLs were measured by real-time PCR. mRNA levels of the *Ppard*^*+/+*^ sample are assigned a value of 1. Data (mean ± SD) represent three independent experiments with similar results. *, *P* < 0.05. **G,** The mRNA levels of indicated genes in human CTLs treated with two different PPARδ siRNAs were measured by real-time PCR. mRNA levels of CTLs treated with the s iC are assigned a value of 1. Data (mean ± SD) represent three independent experiments with similar results. *, *P* < 0.05; **, *P* < 0.02. **H,** The mRNA levels of indicated genes in human CTLs transfected with a plasmid expressing GFP or human PPARδ were measured by real-time PCR. Data (mean ± SD) represent three independent experiments with similar results. Unpaired Student *t* test was used for statistics. *, *P* < 0.05; **, *P* < 0.02.

To determine how PPARδ controls CTL effector function, we first examined its expression in CD8^+^ T cells. Stimulation of naïve CD8^+^ T cells by αCD3/CD28 significantly increased PPARδ expression, and then exposure to IL2 containing medium led to another boost in upregulation (Fig. 2A; Supplementary Fig. S2A).

We then investigated if PPARδ regulates molecules that mediate CTL cytotoxicity (28). After coculture of *Ppard*^*−/−*^ CTLs with cancer cells, we observed higher granzyme B levels in cancer cells than those cocultured with wild-type CTLs (Fig. 1D), suggesting that *Ppard*^*−/−*^ CTLs express more granzyme B because cancer cells do not express granzyme B. Indeed, *Ppard*^*−/−*^ CTLs showed increased expression of granzyme B, perforin, and secreted IFNγ compared with wild-type cells (Fig. 2B; Supplementary Fig. S2B). Overexpression of PPARδ in CTLs reduced the expression of these proteins in both *Ppard*^*−/−*^ and wild-type CTLs (Fig. 2C; Supplementary Fig. S2C). GW501516 treatment reduced the expression of these proteins in *Ppard*^+/+^ but not in *Ppard*^*−/−*^ CTLs, demonstrating GW501516’s specificity (Fig. 2D; Supplementary Fig. S2D). In human CTLs, PPARδ knockdown using two different siRNAs led to increased levels of these proteins, whereas overexpression decreased them (Fig. 2E; Supplementary Fig. S2E). GW501516 or GW0742 treatment also reduced these protein levels (Supplementary Fig. S2F). The upregulation of PGC1α and CPT1A, both of which are PPARδ-regulated, confirms PPARδ activation by these agonists (Supplementary Fig. S2F).

To understand how PPARδ downregulates perforin, granzyme B, and IFNγ, we performed real-time PCR. The mRNA levels of all three genes (*Prf1*, *Gzmb*, and *Ifng*) were significantly higher in *Ppard*^*−/−*^ CTLs than that in wild-type cells (Fig. 2F), whereas *Tnfa* remained unchanged (Fig. 2F). In human CTLs, PPARδ inhibition by siRNAs increased mRNA levels of these genes, whereas overexpression decreased them (Fig. 2G and H). GW501516 or GW0742 treatment also reduced mRNA levels of these genes (Supplementary Fig. S2G). These results indicate that PPARδ may regulate the transcription of perforin, granzyme B, and IFNγ.

### PPARδ binds to RelA in the nucleus, interfering with RelA–p50 heterodimer formation in CTLs

**Figure 3:**
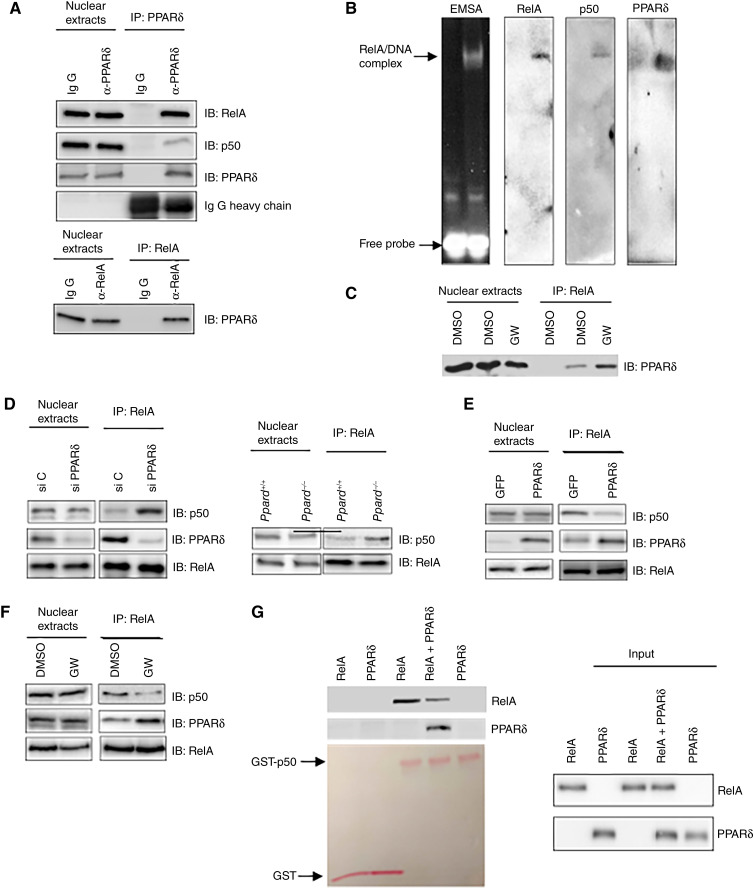
PPARδ binds RelA and interferes with the RelA/p50 heterodimer formation. **A,** Interaction of RelA with PPARδ in nuclear extracts of human CTLs was revealed by two-way coimmunoprecipitations. **B,** RelA, p50, and PPARδ form a κB site-bound protein complex in human CTL nuclear extracts was revealed by EMSA and Western blot. **C,** Treating human CTLs with GW501516 (GW) enhances the interaction between RelA and PPARδ. **D,** PPARδ deficiency enhances the binding of p50 to RelA in human and mouse CTLs. **E,** Overexpression of PPARδ in human CTLs reduces the binding of p50 to RelA. **F,** Treatment of human CTLs with GW501516 (GW) reduces the binding of p50 to RelA. The results from **C–F** were obtained from coimmunoprecipitation assays. **G,** Reduced RelA binding to p50 in the presence of PPARδ in GST pulldown assay using purified proteins. Data are representative of three independent experiments with similar results.

PPARδ can bind to PPREs within gene promoters to regulate transcription. However, a computer sequence analysis did not identify potential PPREs in the promoters (2 kb upstream of the transcription starting site) of *Gzmb* and *Ifng*, implying an indirect mechanism. All three genes are known to be directly regulated by the NF-κB family member RelA (19, 20, 25). Previous reports show that PPARδ can inhibit NF-κB activation by physically interacting with RelA (29, 30). Using a CHIP–qPCR assay, we found that both PPARδ and RelA bound to the regions near the transcription start site in the promoters of all three genes (Supplementary Fig. S3A and S3B), suggesting a direct interaction. Co-immunoprecipitation confirmed that PPARδ is part of a protein complex with RelA and NF-κB1/p50 in the nucleus of CTLs (Fig. 3A). This protein complex binds to a consensus NF-κB DNA probe *in vitro* (Fig. 3B). GW501516 treatment facilitated PPARδ binding to RelA in the nucleus (Fig. 3C; Supplementary Fig. S4A). PPARδ knockdown with siRNAs enhanced p50 binding to RelA in human CTLs [Fig. 3D (left); Supplementary Fig. S4B]. Similarly, more p50 was associated with RelA in *Ppard*^*−/−*^ cells than in *Ppard*^*+/+*^ cells [Fig. 3D (right); Supplementary Fig. S4B]. Overexpression of PPARδ (Fig. 3E; Supplementary Fig. S4C) or GW501516 treatment (Fig. 3F; Supplementary Fig. S4D) inhibited p50 binding to RelA. A GST pulldown assay showed that PPARδ inhibited the binding of RelA to GST-tagged p50 but did not bind GST-p50 itself (Fig. 3G; Supplementary Fig. S4E). Control experiments confirmed that neither RelA nor PPARδ interacted with GST protein (Fig. 3G). These results demonstrate that PPARδ interferes with RelA/p50 heterodimer formation.

### PPARδ downregulates RelA DNA binding/transcriptional activity in CTLs

**Figure 4:**
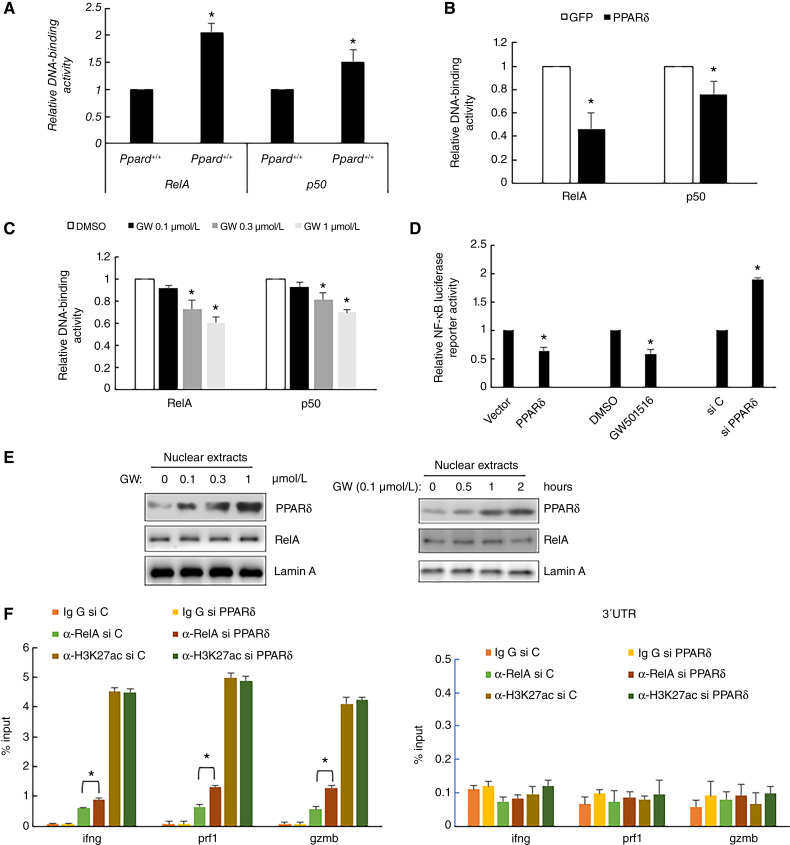
PPARδ inhibits RelA/p50 DNA-binding activity. **A–C,** DNA-binding activity of RelA and p50 in the nucleus of *Ppard*^*+/+*^ and *Ppard*^*−/−*^ murine CTLs (**A**), human CTLs transfected with plasmid expression GFP or human PPARδ (**B**), human CTLs treated with indicated concentrations of GW501516 (GW; **C**). Data (mean ± SD) represent three independent experiments with similar results. *, *P* < 0.05. **D,** Reporter activity of an NF-κB luciferase reporter in human CTLs transfected with a plasmid expressing PPARδ, treated with GW501516, or treated with PPARδ siRNA. **E,** Western blot expression of PPARδ and other indicated proteins in the nuclear extracts of human CTLs treated with GW501516 (GW). **F,** Binding of RelA to the promoters of *Ifng, Gzmb*, or *Prf1* gene in human CTLs treated with PPARδ siRNA in CHIP–qPCR assays. Primer pairs amplify regions close to the transcription start sites using a control IgG antibody or antibody against RelA or acetyl-Histone H3 (Lys27). CHIP–qPCR data from these samples using the 3′-UTR primer pairs are shown. Data (mean ± SD) represent three independent experiments with similar results. *, *P* < 0.05.

RelA and p50 DNA-binding activities are higher in *Ppard*^*−/−*^ CTLs (Fig. 4A) and in human CTLs treated with PPARδ siRNA (Supplementary Fig. S5A) than in their corresponding control cells. Overexpression of PPARδ (Fig. 4B) or adding recombinant PPARδ (Supplementary Fig. S5B) suppressed RelA or p50 DNA-binding activities. GW501516 treatment of CTLs also reduced these activities (Fig. 4C). In NF-κB-luciferase reporter assays, PPARδ overexpression or GW501516 treatment decreased firefly luciferase activity. In contrast, PPARδ knockdown increased it (Fig. 4D). Luciferase activity was also higher in *Ppard*^*−/−*^ CTLs compared with *Ppard*^*+/+*^ cells (Supplementary Fig. S5C). GW501516 treatment led to a dose- and time-dependent nuclear translocation of PPARδ but not RelA in both human (Fig. 4E; Supplementary Fig. S5D) and murine (Supplementary Fig. S5E) CTLs . Because PPARδ did not affect the DNA-binding activity of purified RelA *in vitro* (Supplementary Fig. S5F), we hypothesize that PPARδ disrupts RelA/p50 heterodimer formation, thus inhibiting its DNA-binding activity. CHIP–qPCR assays showed that PPARδ siRNA knockdown increased RelA binding to the promoters of *Prf1*, *Gzmb*, or *Ifng* genes. The siRNA knockdown of PPARδ increased the binding of RelA to the promoters of all three genes (Fig. 4F). Conversely, GW501516 treatment (Supplementary Fig. S5G) or PPARδ overexpression (Supplementary Fig. S5H) reduced RelA binding to the promoters of all three genes.

### RelA, IFNγ, and granzyme B are critical for the cytotoxicity of CTLs

**Figure 5:**
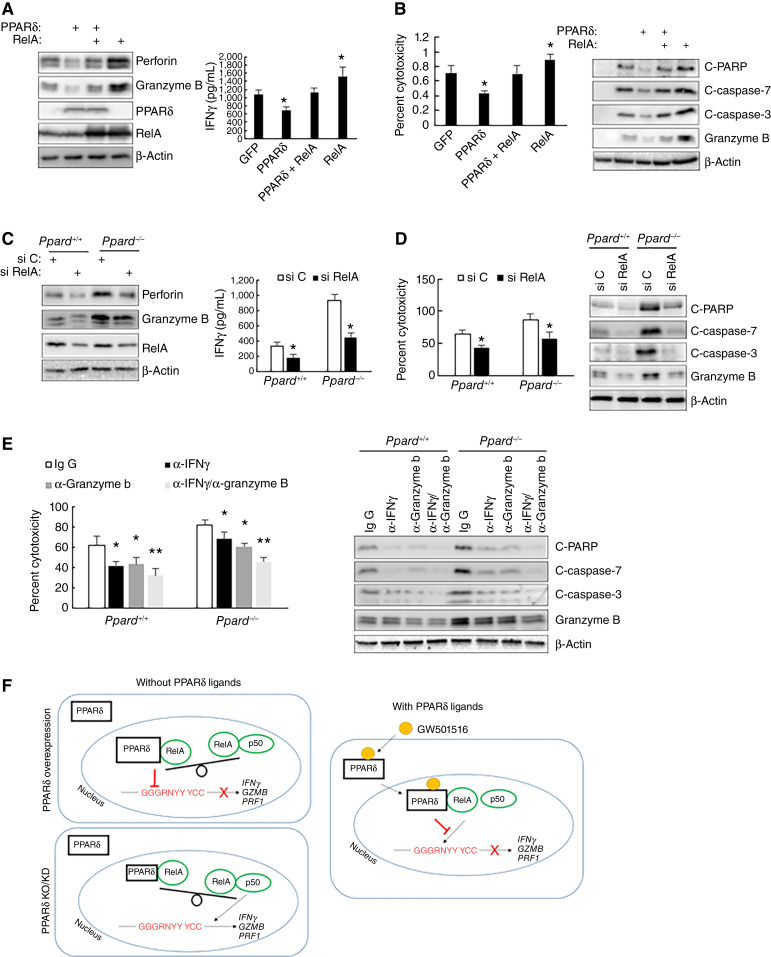
RelA, IFNγ, and granzyme B are critical for CTL cytolytic activity. **A,** Western blot expression of indicated proteins in human CTLs transfected with a plasmid expressing RelA or PPARδ and ELISA expression of IFNγ in the culture media of the CTLs. Data (mean ± SD) represent three independent experiments with similar results. *, *P* < 0.05. **B,** The cytotoxicity of human CTLs (18-hour incubation, left) and Western blot expression of indicated proteins in HCT116 cells (2-hour incubation, right) after the CTLs transfected with plasmid expressing PPARδ or human RelA were incubated with HCT116 cells. Data (mean ± SD) represent three independent experiments with similar results. *, *P* < 0.05. **C,** Western blot expression of indicated proteins in *Ppard*^*+/+*^ and *Ppard*^*−/−*^ murine CTLs transfected with a control siRNA (si C) or siRNA targeting RelA (si RelA) and ELISA expression of IFNγ in the culture media of the CTLs. Data (mean ± SD) represent three independent experiments with similar results. *, *P* < 0.05. **D,** The cytotoxicity of *Ppard*^*+/+*^ and *Ppard*^*−/−*^ murine CTLs (18-hour incubation, left) and Western blot expression of indicated proteins in HCT116 cells (2-hour incubation, right) after *Ppard*^*+/+*^ and *Ppard*^*−/−*^ CTLs transfected with a control siRNA (si C) or siRNA targeting RelA (si RelA) were incubated with HCT116 cells. Data (mean ± SD) represent three independent experiments with similar results. *, *P* < 0.05. **E,** The cytotoxicity of human CTLs (18-hour incubation, left) and Western blot expression of indicated proteins in HCT116 cells (2-hour incubation, right) after the CTLs treated with a control antibody (Ig G) or antibody against IFNγ (α-IFNγ), or granzyme B (α-GZMB), or the combination of the two antibodies were incubated with HCT116 cells. Data (mean ± SD) represent three independent experiments with similar results. *, *P* < 0.05; **, *P* < 0.02. **F,** A hypothetical model of PPARδ interactions with RelA in the nucleus of CTLs. C-PARP, cleaved PARP; C-caspase 3, cleaved caspase 3; C-caspase 7, cleaved caspase 7.

Overexpression of RelA increased the expression of perforin, granzyme B, and IFNγ in CTLs (Fig. 5A; Supplementary Fig. S6A), whereas RelA knockdown using siRNA reduced their expression (Fig. 5B; Supplementary Fig. S6B), confirming these genes are RelA targets. RelA overexpression also enhanced CTL cytotoxicity toward tumor cells (Fig. 5C; Supplementary Fig. S6C), whereas RelA knockdown decreased it (Fig. 5D; Supplementary Fig. S6D), highlighting RelA’s critical role in CTL cytotoxicity. Overexpression of PPARδ attenuated the effects of RelA overexpression on the expression of these genes, indicating dynamic interactions between PPARδ and RelA (Fig. 5A). PPARδ overexpression alone also decreased the expression of perforin, granzyme B, and IFNγ (Fig. 5A). Neutralizing antibodies against IFNγ or granzyme B suppressed CTL cytotoxicity and reduced apoptotic markers in tumor cells. Combined treatment with both antibodies had more effect on CTL cytotoxicity than any single antibody alone (Fig. 5E; Supplementary Fig. S6E). These results demonstrate the importance of IFNγ and granzyme B in regulating CTL cytotoxicity.

## Discussion

After reviewing the results of previous studies, we concluded that the specific effects of RelA-mediated gene regulation on T-cell immunity needed to be clarified with regard to PPARδ. Both PPARα and PPARγ can interfere with RelA’s transcriptional activity (10). For the first time, we provide direct evidence that PPARδ inhibits CTL cytotoxicity by downregulating RelA DNA-binding activity, thereby reducing the expression of perforin, granzyme B, and IFNγ. This suggests that PPARδ ligands have the opposite effect on T-cell therapy compared with PPARα and PPARγ ligands, supporting a protumorigenic role for PPARδ in immune cells. Despite some controversy, most published studies indicate that PPARδ significantly contributes to tumorigenesis in several cancers (reviewed in ref. 31, 32).

Our study revealed that PPARδ competes with p50 for binding to RelA in the nucleus of CTLs, acting as a transrepressor (Fig. 5F). Ligand-bound cytosolic PPARδ translocates to the nucleus, where it disrupts the RelA–p50 interaction (Fig. 5F). CTLs can kill tumor cells through at least three distinct pathways (28). In direct cell–cell contact, the CTLs release lytic granules containing perforin and granzymes into the intercellular space of tumor cells, leading to cell death in a caspase-dependent and -independent manner (33). Alternatively, cell killing can be mediated by cytokines secreted by CTLs, like IFNγ and TNFα. Our *in vivo* study shows that GW501516 treatment reduces tumor-associated CD8^+^ T-cell activation, as evidenced by decreased proliferation and IFNγ expression (Fig. 1A and B). Our *in vitro* results show that *Ppard*^*−/−*^-CD8^+^ T cells secreted more IFNγ than *Ppard*^*+/+*^-CD8^+^ T cells (Fig. 2C), indicating that PPARδ modulates CD8^+^ T-cell activation and subsequent effector function.

PPARδ affects NF-κB at multiple levels, including (i) inhibiting nuclear translocation of RelA in rat heart tissue (34), (ii) reducing RelA acetylation in human HaCaT keratinocytes (35), and (iii) interacting with RelA in a ligand-dependent manner in microglia (29) and cardiomyocytes (30). Our study shows that nuclear PPARδ binds to RelA in the absence of ligand, and this interaction is enhanced following ligand treatment of human and mouse CD8^+^ T cells (Fig. 3). PPARδ competes with p50 for RelA binding. Because PPARδ does not affect the DNA-binding activity of purified p65 or p50 *in vitro* (Supplementary Fig. S4E), we postulated that PPARδ disrupts RelA/p50 dimer formation, reducing its DNA-binding activity. This dimer has the highest affinity for NF-κB sites and transcriptional activity compared with all other NF-κB dimers (36). These findings enhance our understanding of the regulation of RelA by PPARδ and provide insight into the molecular mechanism by which PPARδ modulates genes controlled by RelA in CD8^+^ T cells. Identifying other RelA target genes regulated by PPARδ in CD8^+^ T cells and determining if PPARδ interferes with other RelA heterodimers will further elucidate PPARδ’s role in CD8^+^ T cells. Not all RelA-targeted genes expressed in CD8^+^ T cells are regulated by PPARδ (data not shown), suggesting it only controls a specific subset of genes in CTLs.

The modulation of CTL cytotoxicity by PPARδ presents a new opportunity for cancer immunotherapy. Advances in checkpoint inhibitors and genetically modified immune cells have shifted our thinking about cancer treatment toward mobilizing the host’s immune system to target cancer cells (37). Despite significant success, many patients do not benefit from these therapies (primary resistance), and some responders relapse (acquired resistance). Enhancing endogenous T-cell function is being explored to combat resistance. PPARδ’s ability to reduce CTL cytotoxicity makes it a potential target for combination therapies to improve CTL effector function. However, PPARδ’s roles vary depending on the context (e.g., healthy vs. diseased tissues; ref. 32). PPARδ can help normal cells endure metabolic challenges, and its agonists may be used to treat metabolic syndrome-associated abnormalities (7). Future therapeutic agents targeting PPARδ must be carefully designed to avoid risks and off-target effects, primarily on normal cells. Chimeric antigen receptor T-cell therapy may benefit from targeting PPARδ to enhance T-cell function, but using PPARδ antagonists requires careful evaluation, as ligand-free PPARδ is functional. PPARδ could also be a challenging target because it regulates the expression of many genes by different mechanisms. For example, PPARδ agonists GW501516 and GW0742 have been shown to inhibit CTL cytolytic activity but also induce the expression of PGC1α (supplementary Fig. S2A) that promotes fatty acid oxidation (38), both of which may impact CD8^+^ T-cell antitumor immunity (39–41).

Here, we report a novel function of PPARδ in CTLs. Our understanding of PPARδ’s interactions with its endogenous ligands, lipid transporters, other nuclear receptors, coactivators, and repressors remains incomplete. Gaining detailed knowledge of PPARδ’s actions in CTLs and other immune cell types will help elucidate the molecular mechanisms by which CTLs eradicate cancer cells and aid the development of new therapeutic strategies, possibly both for cancer prevention/interception and treatment.

## Supplementary Material

Supplementary Fig. 1 Supplementary Fig. 1 shows PPARδ inhibits CD8+ T cell cytolytic activity.

Supplementary Fig. 2 Supplementary Fig. 2 shows PPARδ negatively regulates the expression of perforin, granzyme B, and IFNγ.

Supplementary Fig. 3 Supplementary Fig. 3 shows RelA and PPARδ bind to the same DNA fragments of IFNγ, granzyme B, or perforin gene promoters in human CTLs.

Supplementary Fig. 4 Supplementary Fig. 4 shows the densitometric analysis of western blot results presented in Fig. 3.

Supplementary Fig. 5 Supplementary Fig. 5 shows PPARδ inhibits RelA/p50 DNA binding activity.

Supplementary Fig. 6 Supplementary Fig. 6 shows the densitometric analysis of western blot results presented in Fig. 5.
