# Supplementary material for: Peroxisome Proliferator–Activated Receptor δ Suppresses the Cytotoxicity of CD8+ T Cells by Inhibiting RelA DNA-Binding Activity
Source: Cancer Res Commun. 2024 Oct 14;4(10):2673–84. doi: 10.1158/2767-9764.CRC-24-0264 (PMC11471967; doi:10.1158/2767-9764.CRC-24-0264)
Supplement: Supplementary Fig. 1 — shows PPARδ inhibits CD8+ T cell cytolytic activity. [file crc-24-0264_supplementary_fig.1_suppsf1.pdf]

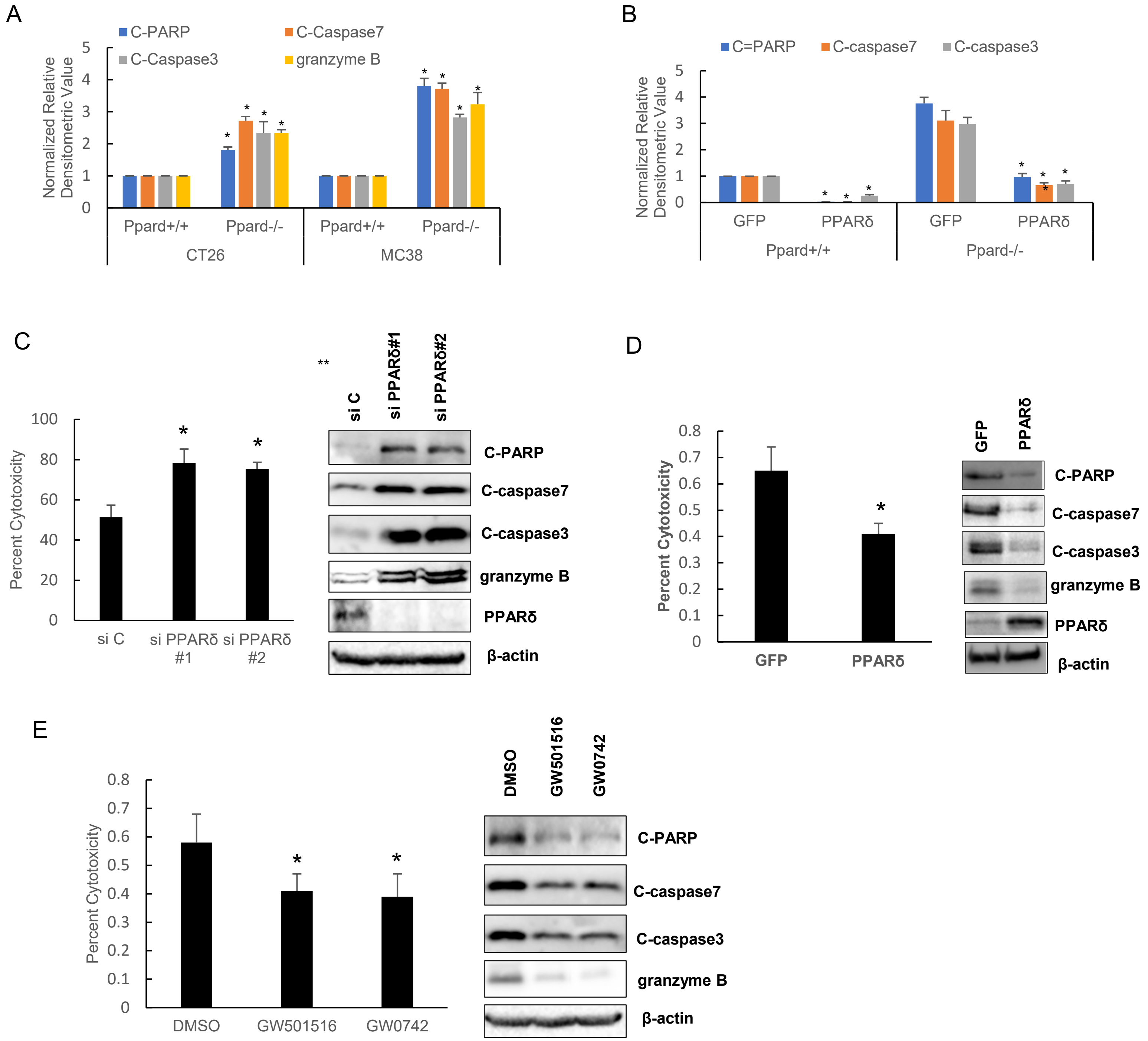

Supplementary Figure 1. PPARδ inhibits CD8^{+} T cell cytolytic activity. (A, B) Densitometric analysis of western blot results presented in Fig. 1D and Fig. 1E. Western blots were normalized to β –actin and densitometric analysis was performed using image processing software ImageJ. Values are mean ± standard error of the mean of at least three independent experiments. *P<0.05, **P<0.02 with comparisons were with (A) Ppard+/+, (B) GFP. Human CTLs were treated with two different PPARδ siRNAs (C) or transfected with a plasmid expressing GFP or human PPARδ (D) or treated with DMSO or GW501516 or GW0742 (E). The cytotoxicity of CTLs (18-hour incubation, left panel) and Western Blot expression of indicated proteins in HCT116 cells (2-hour incubation, right panel) are shown. Data (mean ± SD) represent three independent experiments with similar results. *p < 0.05.
