# Supplementary material for: Peroxisome Proliferator–Activated Receptor δ Suppresses the Cytotoxicity of CD8+ T Cells by Inhibiting RelA DNA-Binding Activity
Source: Cancer Res Commun. 2024 Oct 14;4(10):2673–84. doi: 10.1158/2767-9764.CRC-24-0264 (PMC11471967; doi:10.1158/2767-9764.CRC-24-0264)
Supplement: Supplementary Fig. 2 — shows PPARδ negatively regulates the expression of perforin, granzyme B, and IFNγ. [file crc-24-0264_supplementary_fig.2_suppsf2.pdf]

Supplementary Fig. 2

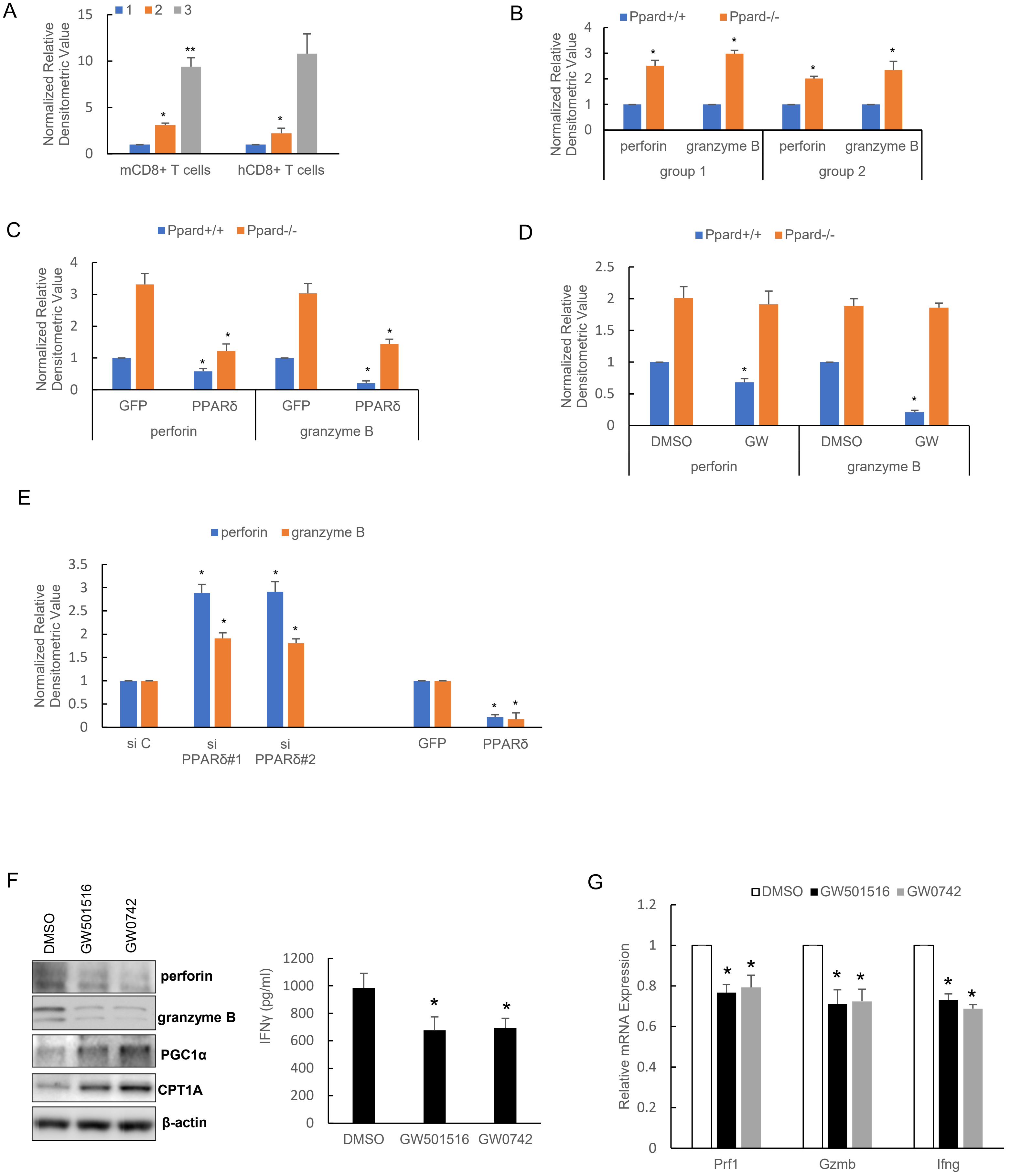

Supplementary Figure 2. PPARδ negatively regulates the expression of perforin, granzyme B, and IFNγ. (A, B. C. D, E) Densitometric analysis of western blot results presented in Fig. 2A, Fig. 2B, Fig. 2C, Fig. 2D, and Fig. 2D, respectively. Western blots were normalized to β –actin and densitometric analysis was performed using image processing software ImageJ. Values are mean ± standard error of the mean of at least three independent experiments. *P<0.05, **P<0.02 with comparisons were with (A) 1, (B) Ppard+/+, (C) GFP, (D) DMSO, (E) si C, GFP. (F) Western blot expression of indicated proteins in human CTLs treated with GW501516 or GW0742 at 1 μmol/L and ELISA expression of IFNγ in the culture media of the CTLs. Comparison was with the DMSO sample. Data (mean ± SD) represent three independent experiments with similar results. *$p < 0.05$. (G). The mRNA levels of indicated genes in human CTLs treated with GW501516 or GW0742 at 1 μmol/L were measured by real-time PCR. Data (mean ± SD) represent three independent experiments with similar results. *$p < 0.05$.
