# Supplementary material for: Peroxisome Proliferator–Activated Receptor δ Suppresses the Cytotoxicity of CD8+ T Cells by Inhibiting RelA DNA-Binding Activity
Source: Cancer Res Commun. 2024 Oct 14;4(10):2673–84. doi: 10.1158/2767-9764.CRC-24-0264 (PMC11471967; doi:10.1158/2767-9764.CRC-24-0264)
Supplement: Supplementary Fig. 3 — shows RelA and PPARδ bind to the same DNA fragments of IFNγ, granzyme B, or perforin gene promoters in human CTLs. [file crc-24-0264_supplementary_fig.3_suppsf3.pdf]

A

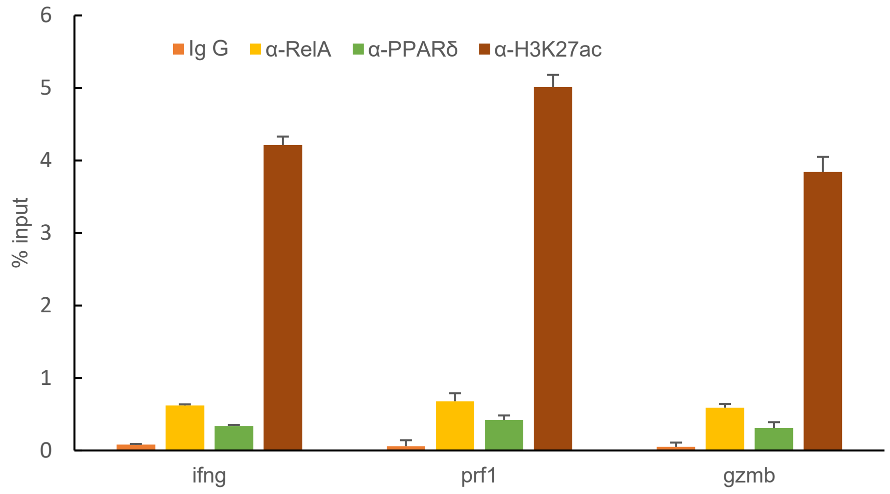

B

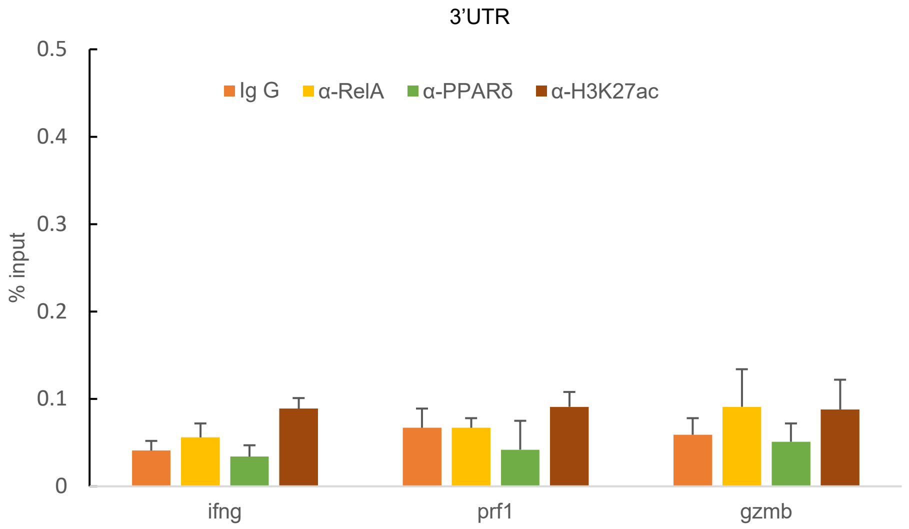

Supplementary Figure 3. RelA and PPARδ bind to the same DNA fragments of IFNγ, granzyme B, or perforin gene promoters in human CTLs. CHIP-qPCR assays targeting the Ifng, Gzmb, or Prf1 gene promoters with primer pairs amplifying regions close to the transcription start sites using a control IgG antibody or antibody against RelA or PPARδ or acetyl-Histone H3 (Lys27) (A). Panel (B) shows CHIP-qPCR data from these samples using the 3'-untranslated region (3'-UTR) primer pairs. Data (mean ± SD) represent three independent experiments with similar results.
