# Supplementary material for: Peroxisome Proliferator–Activated Receptor δ Suppresses the Cytotoxicity of CD8+ T Cells by Inhibiting RelA DNA-Binding Activity
Source: Cancer Res Commun. 2024 Oct 14;4(10):2673–84. doi: 10.1158/2767-9764.CRC-24-0264 (PMC11471967; doi:10.1158/2767-9764.CRC-24-0264)
Supplement: Supplementary Fig. 4 — shows the densitometric analysis of western blot results presented in Fig. 3. [file crc-24-0264_supplementary_fig.4_suppf.pdf]

Supplementary Fig. 4

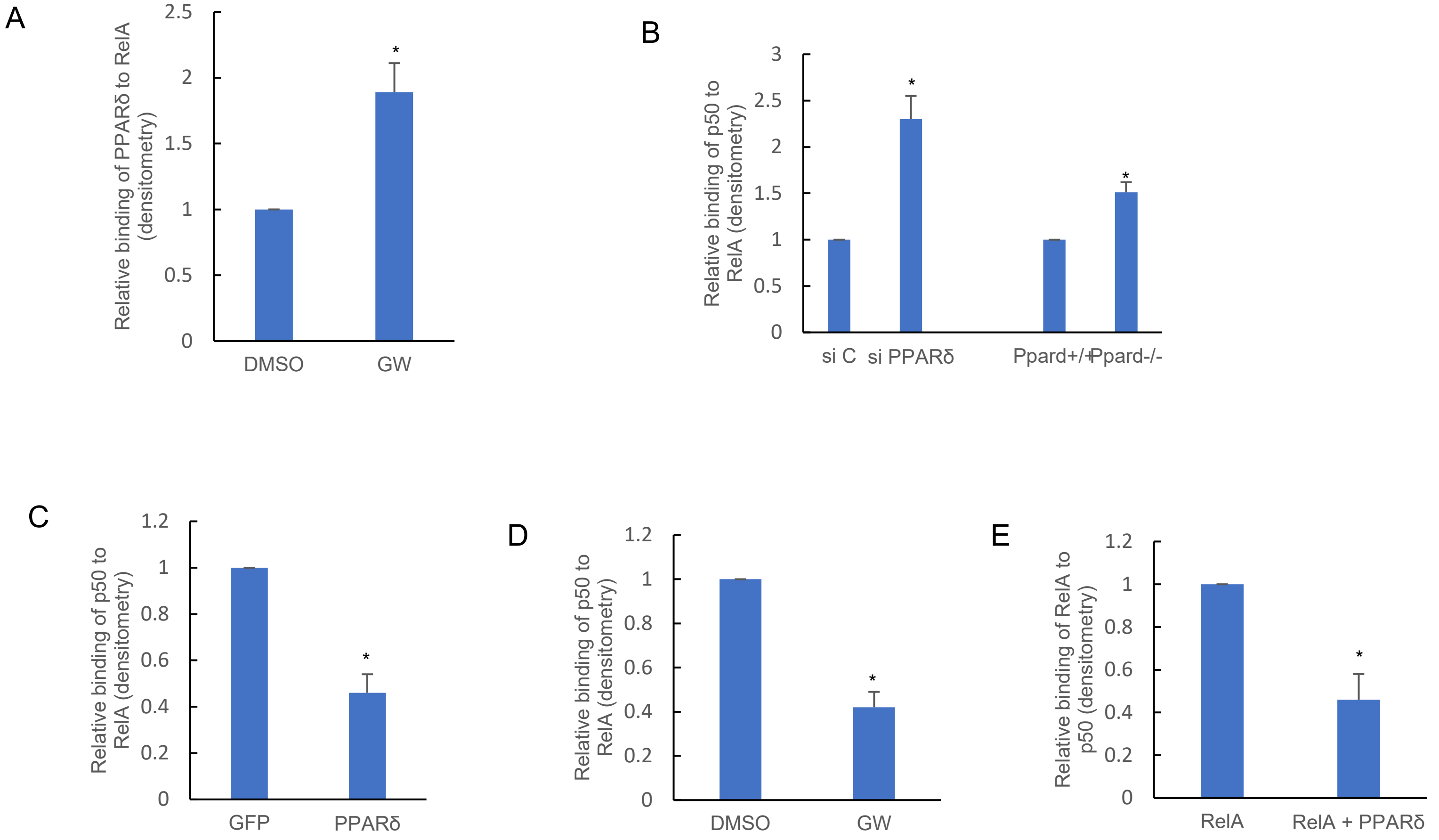

Supplementary Figure 4. Densitometric analysis of western blot results presented in Fig. 3C, Fig. 3D, Fig. 3E, Fig. 3F, and Fig. 3G, respectively. Western blots were normalized to β –actin and densitometric analysis was performed using image processing software ImageJ. Values are mean ± standard error of the mean of at least three independent experiments. *P<0.05, **P<0.02 with comparisons were with (A) DMSO, (B) si C or *Ppard+/+,* (C) GFP, (D) DMSO, (E) RelA.
