# Supplementary material for: Peroxisome Proliferator–Activated Receptor δ Suppresses the Cytotoxicity of CD8+ T Cells by Inhibiting RelA DNA-Binding Activity
Source: Cancer Res Commun. 2024 Oct 14;4(10):2673–84. doi: 10.1158/2767-9764.CRC-24-0264 (PMC11471967; doi:10.1158/2767-9764.CRC-24-0264)
Supplement: Supplementary Fig. 5 — shows PPARδ inhibits RelA/p50 DNA binding activity. [file crc-24-0264_supplementary_fig.5_suppsf5.pdf]

Supplementary Fig. 5

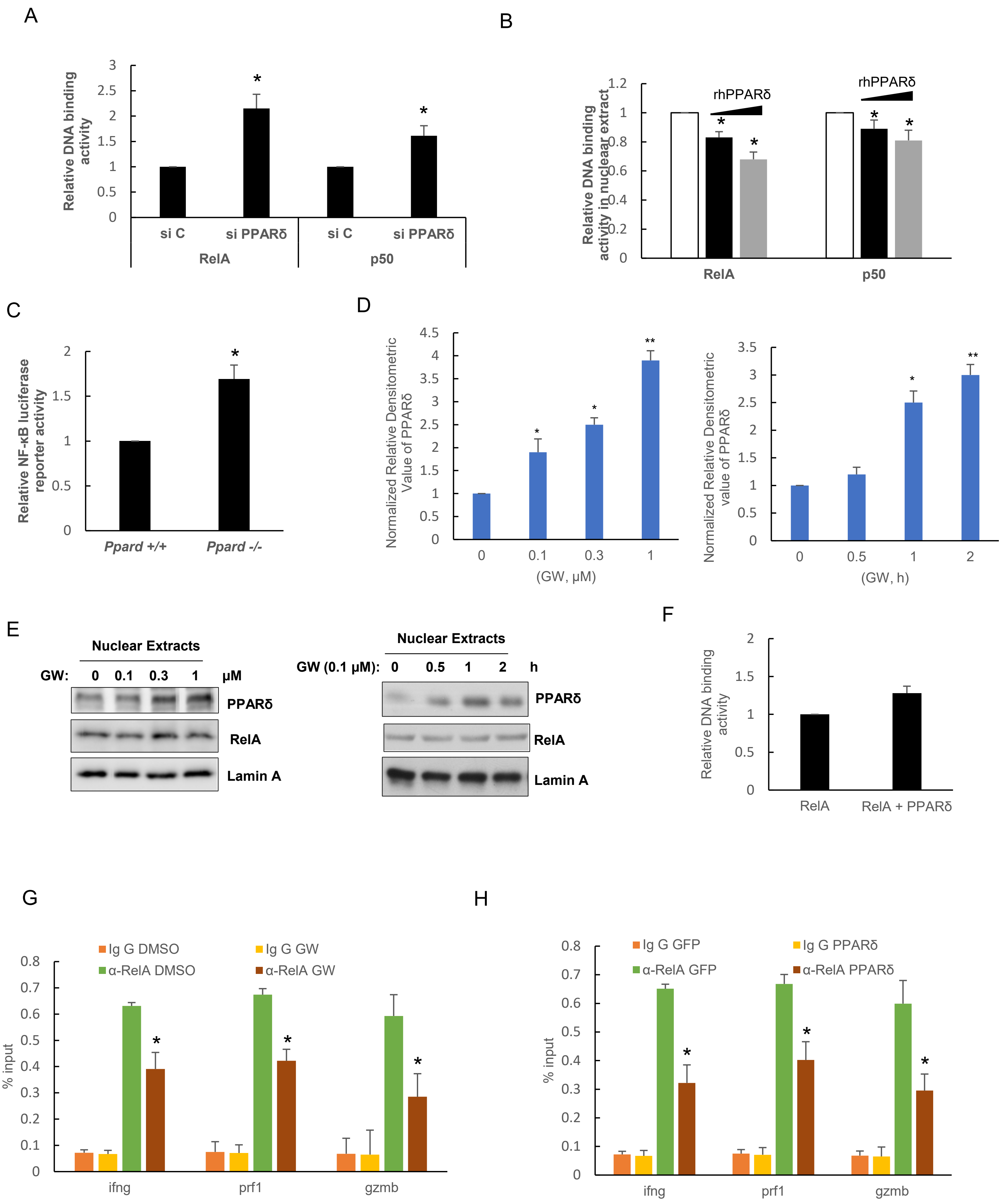

**Supplementary Figure 5**. PPARδ inhibits RelA/p50 DNA binding activity. (A) DNA binding activity of RelA and p50 in the nucleus of human CTLs transfected a control siRNA (si C) or siRNA targeting PPARδ. Data (mean ± SD) represent three independent experiments with similar results. *$p < 0.05$. (B) DNA binding activity of RelA and p50 in the nucleus of human CTLs incubated with recombinant human PPARδ. Data (mean ± SD) represent three independent experiments with similar results. *$p < 0.05$. (C) Reporter activity of an NF-kB luciferase reporter in *Ppard+/+* and *Ppard-/-* murine CTLs. Data (mean ± SD) represent three independent experiments with similar results. *$p < 0.05$. (D) Densitometric analysis of western blot results presented in Fig 4E. Western blots were normalized to β –actin and densitometric analysis was performed using image processing software ImageJ. Values are mean ± standard error of the mean of at least three independent experiments. *$P<0.05$, **$P<0.02$ with comparisons were with 0 μM and 0 h. (E) Western blot expression of PPARδ and other indicated proteins in the nuclear extracts of murine CTLs treated with GW501516 (GW). (F) DNA binding activity of recombinant RelA and p50 in the absence or presence of recombinant PPARδ. (G, H) Binding of RelA to the promoters of Ifng, Gzmb, or Prf1 gene in human CTLs treated with GW501516 or DMSO (G) or transfected with a PPARδ or GFP expressing plasmid (H) in CHIP-qPCR assays. Primer pairs amplify regions close to the transcription start sites using a control IgG antibody or antibody against RelA. Data (mean ± standard deviation) represent three independent experiments with similar results. Data (mean ± SD) represent three independent experiments with similar results. *$p < 0.05$.
